# Rapid Plasticity in the Prefrontal Cortex during Affective Associative Learning

**DOI:** 10.1371/journal.pone.0110720

**Published:** 2014-10-21

**Authors:** Maimu A. Rehbein, Christian Steinberg, Ida Wessing, María Carmen Pastor, Pienie Zwitserlood, Kati Keuper, Markus Junghöfer

**Affiliations:** 1 Institute for Biomagnetism and Biosignalanalysis, University Hospital Münster, Münster, Germany; 2 Otto Creutzfeldt Center for Cognitive and Behavioral Neuroscience, University of Münster, Münster, Germany; 3 Department of Child and Adolescent Psychiatry, University Hospital Münster, Münster, Germany; 4 Department of Psychology, Jaume I University, Castellón, Spain; 5 Institute of Psychology, University of Münster, Münster, Germany; University Medical Center Goettingen, Germany

## Abstract

MultiCS conditioning is an affective associative learning paradigm, in which affective categories consist of many similar and complex stimuli. Comparing visual processing before and after learning, recent MultiCS conditioning studies using time-sensitive magnetoencephalography (MEG) revealed enhanced activation of prefrontal cortex (PFC) regions towards emotionally paired versus neutral stimuli already during short-latency processing stages (i.e., 50 to 80 ms after stimulus onset). The present study aimed at showing that this rapid differential activation develops as a function of the acquisition and not the extinction of the emotional meaning associated with affectively paired stimuli. MEG data of a MultiCS conditioning study were analyzed with respect to rapid changes in PFC activation towards aversively (electric shock) paired and unpaired faces that occurred during the learning of stimulus-reinforcer contingencies. Analyses revealed an increased PFC activation towards paired stimuli during 50 to 80 ms already during the acquisition of contingencies, which emerged after a single pairing with the electric shock. Corresponding changes in stimulus valence could be observed in ratings of hedonic valence, although participants did not seem to be aware of contingencies. These results suggest rapid formation and access of emotional stimulus meaning in the PFC as well as a great capacity for adaptive and highly resolving learning in the brain under challenging circumstances.

## Introduction

Emotions influence the value ascribed to experiences, they aid in selecting the appropriate action, and they guide behavior towards approaching or avoiding specific situations [1]. In line with such a survival-promoting function of emotion, emotional compared to neutral stimuli have been shown to receive preferential neural processing [2]. Functional Magnetic Resonance Imaging (fMRI) and Positron Emission Tomography (PET) studies revealed enhanced neuronal responses towards a variety of different emotional stimuli in a widely distributed neural network, including the amygdala, the prefrontal cortex (PFC), and the sensory cortices (e.g., [3]–[5]). Temporally high-resolution event-related potential (ERP) and event-related magnetic field (ERMF) studies indicated distinct modulations of brain responses by emotional pictures during mid-latency (i.e., 120–300 ms) and late (i.e., >300 ms) processing stages [6]–[9]. Importantly, visually presented emotional stimuli may influence neuronal responses much earlier in the processing stream than previously assumed. For example, aversively conditioned simple geometric shapes altered visual ERP components in the C1 time interval between 60 and 90 ms following stimulus presentation [10]–[12]. Perceptually complex emotional stimuli, such as emotional faces, also produced differential effects on short-latency components [13]–[15].

Furthermore, recent studies using *MultiCS conditioning* revealed the involvement of PFC regions in the rapid processing of emotional stimuli [16]. In MultiCS conditioning studies, many neutral and perceptually similar stimuli (CS+) are paired with either a single or a multitude of affective unconditioned stimuli (US). The same number of similar CS stimuli (CS−) are paired with a single or a multitude of neutral (non affective) US or are not paired at all. Via this procedure, the CS+ adopt the emotional value of the affective US, while the CS− remain neutral. This novel associative learning paradigm thus creates multiple conditioned stimuli that differ in their emotional valence, but that are similar in their perceptual properties. Due to the multitude of similar stimuli and the limited contingency information available (i.e., MultiCS conditioning studies have never included more than four learning trials for each CS), MultiCS conditioning poses a highly demanding situation in which the brain's learning capacity is challenged. It has been assumed that the high task demands increase the probability of higher-level brain areas being involved, which is why MultiCS conditioning seems especially suited to reveal prefrontal participation in the rapid processing of emotional stimuli (see [16], for more information concerning this paradigm). Indeed, a visuo-olfactory MultiCS conditioning study [17] reported greater cortical activation after learning in prefrontal and sensory areas towards faces paired with an aversive odor compared to faces paired with a neutral odor. The enhanced CS+ activation was present during an early (i.e., 50–80 ms) and a mid-latency (i.e., 130–190 ms) time interval. An auditory-auditory MultiCS conditioning study [18] revealed increased neuronal activation after learning in prefrontal and temporal areas towards click-tones paired with aversive or appetitive sounds relative to click-tones paired with neutral sounds. The differential CS activation emerged during the N1m time interval. In both studies, enhanced short-latency responses to CS+ vs. CS− recorded in the PFC after learning were interpreted as signaling preferential processing of emotional stimuli.

However, it remains unclear whether the increase in rapid prefrontal activation recorded after learning truly arises due to the access of the emotional value of the CS+. So far, MultiCS conditioning studies have investigated the learning-induced changes in stimulus processing only by comparing CS+/CS− evoked neural activity after versus before learning. Stimulus processing during learning has not yet been examined. Since measurements after learning were based on unreinforced presentations of the previously paired stimuli, enhanced PFC activation could also point towards extinction processes, instead of indicating access of CS+/US representations formed during learning. Indeed, a great number of classical conditioning studies in humans and rodents (e.g., [19], [20]) point towards an activation of the PFC especially during the extinction phase of conditioning, which involves learning of new CS+/noUS associations that diminish the expression of fear. The present study investigated whether enhanced PFC activation towards emotionally paired stimuli observed after learning in a MultiCS conditioning setting indicates the access of CS+/US representations formed during learning or whether it reflects extinction-related processes. In addition, we applied a non-parametric analysis method in which multiple testing was strictly controlled for to examine the reliability of enhanced PFC activation after learning. Previous MultiCS conditioning studies have so far employed parametric analysis methods in which the probability of a type-I error after multiple testing was reduced by limiting the analysis to those effects which were present in a minimum number of neighboring sensors or dipoles (i.e., in a spatially extended cluster) and a minimum number of adjacent time-points (i.e., in a longer time interval).

Therefore, we analyzed the acquisition phase and reanalyzed the pre- and post-learning phases of one MultiCS conditioning study, in which multiple neutral faces were paired with an aversive electric stimulation to the right or the left hand, while the same number of neutral faces remained unpaired. In this study, whole-head magnetoencephalography (MEG) revealed enhanced activation towards paired vs. unpaired faces in the PFC during 50 to 80 ms after learning (as published in [16]), consistent with the visuo-olfactory MultiCS conditioning study [17]. For the article at hand, we tested whether this enhanced rapid PFC activation would survive a more conservative analysis procedure. Furthermore, we investigated whether the early differential PFC activation observed after learning developed during the acquisition phase, which would argue against an extinction-centered interpretation of the short-latency effects. Additional subjective and behavioral measures assessed whether the challenging MultiCS conditioning procedure employed here could influence affective ratings and action tendencies and whether it would occur in the absence of contingency awareness.

We focused on early affective processing in the PFC and did not investigate later components of affective processing, so that we could discuss rapid affective discrimination in MultiCS conditioning with the required detail. Parts of the results reported here (including the prefrontal differentiation before vs. after learning and the behavioral indices of emotional learning) have briefly been included in a review about MultiCS conditioning [16]. To provide a more detailed discussion, these previously reported results were also described here.

## Materials and Methods

### Participants

Forty-eight healthy right-handed individuals (24 female) took part in the study. All participants were native German speakers, had a mean age of 24.5 years (*SD* = 2.9), and normal or corrected-to-normal vision. Before participation, all individuals received written and oral information about the procedure and gave informed written consent to the protocol approved by the ethics committee of the Medical Faculty, University of Muenster (2007-563-f-S). Participants received 30 € for participation.

### Stimuli

#### Conditioned stimuli

One hundred and four images displaying Caucasian faces (52 female) with neutral expression from frontal view were used as conditioned stimuli (CSs). Faces were taken from the Karolinska Directed Emotional Faces archive [21], the NimStim set of facial expressions [22], the picture pool of the Institute for Biomagnetism and Biosignalanalysis, and the FERET database of facial images [23], . Using Adobe Photoshop, faces were adjusted to a height of 15 cm and a resolution of 72 pixels/inch and were converted to gray scale images. Stimuli were pseudo-randomly split into CS+ faces (paired with electric stimulation during conditioning) and CS− faces (unpaired during conditioning). The assignment of pictures to these two conditions was balanced between participants. For the practice trials in the behavioral assessment, 15 additional pictures were used as test images.

#### Unconditioned stimulus

An electric shock consisting of a 300 ms train of 0.5 ms pulses at a rate of 64 Hz served as unconditioned stimulus (US). For half of the CS+ within each block, the US was delivered to the tip of the right index finger, for the other half, to the left index finger. Shock intensity was set for every participant individually to be *highly unpleasant, but not painful*. The US was delivered using a Grass Instruments S-88 dual-channel square-pulse stimulator with an Isolation Unit SIU7 (all by Grass Instrument Division, Astro-Med Inc., West Warwick, RI, USA).

### Experimental procedure

**Figure 1:**
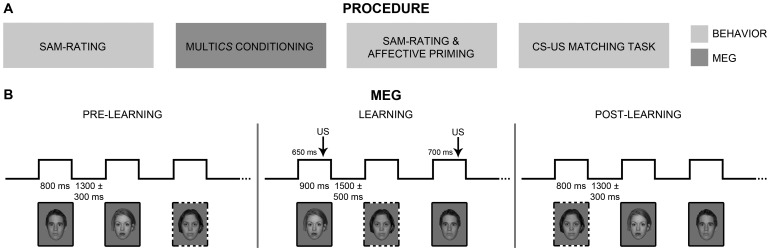
Paradigm. (A) The procedure consisted of assessments of behavior and of neuronal activity via Magnetoencephalography (MEG). For behavior, participants completed subjective (SAM) ratings of hedonic valence and emotional arousal of all conditioned stimuli (CSs) before and after conditioning as well as an affective priming and a CS-US matching task. MEG recordings were acquired, while participants underwent MultiCS conditioning. (B) MultiCS conditioning consisted of three phases: a pre-learning, a learning, and a post-learning phase. During the learning phase, half of the CS faces were paired (CS+; solid frame) with an aversive electric stimulation (US), while the other half remained unpaired (CS−; dashed frame). During pre- and post-learning phases, all CSs were shown without US presentations.

The experiment consisted of three subjective (evaluative) and behavioral tests administered before and after MultiCS conditioning as well as of a measurement of neuronal activation conducted during MultiCS conditioning (Figure 1A). The subjective and behavioral measurement took place outside the MEG scanner, while neuronal activation was measured inside. The MEG scanner was located in a sound attenuated and magnetically shielded room to prevent interference from outside sources.

#### Subjective ratings of valence and arousal

Prior to conditioning, participants completed subjective ratings of hedonic valence and emotional arousal for all CSs, using a computerized version of the Self-Assessment-Manikin (SAM) scale [25], [26]. The original 9-point Likert scales were modified to analogous scales, so that even small deviations in the ratings could be registered. The scales ranged from −300 (*unpleasant*) to +300 (*pleasant*) for hedonic valence and from −300 (*calm*) to +300 (*arousing*) for emotional arousal. Before each rating, participants viewed each face for 900 ms, preceded by a fixation cross, shown for 600 ms. For practice, they completed three test trials.

#### MEG measurement

In preparation of the MEG measurement, information about the individual head shape was acquired using a Polhemus 3Space Fasttrack. Subsequently, participants were comfortably seated in the MEG scanner and completed the pre-learning phase of MultiCS conditioning (Figure 1B). During this phase, all CSs were shown three times in randomized order with an inter-stimulus interval (ISI) of 1300±300 ms. Each CS was presented with a visual angle of about 13° in the center of the screen and a duration of 800 ms. A small fixation cross appeared in the center of the screen during ISI. To minimize ocular artifacts, participants were asked to keep their eyes focused on the center of the screen. After the pre-learning phase, electrodes for electric stimulation were positioned on the tip of the index fingers of the participant’s right and left hand. Participants received a mild electric stimulation (1 mA) and were asked to rate the intensity of the stimulation on a visual rating scale. The scale ranged from 1 to 6, and poles and gradations were labeled as 1 (*not perceptible*), 2 (*slightly perceptible*), 3 (*clearly perceptible, but not unpleasant*), 4 (*slightly unpleasant*), 5 (*highly unpleasant, but not painful*), and 6 (*painful*). Stimulus intensity was steadily increased until participants described it as being *highly unpleasant, but not painful*. After the first and the second acquisition run, participants retrospectively evaluated the shock intensity on the visual rating scale to avoid habituation effects to the US. If necessary, stimulus intensity was again increased up to a *highly unpleasant, but not painful* self-assessment before the next learning run. The learning phase was carried out in three consecutive runs. During each run, participants were shown all CSs once, with a presentation time of 900 ms and an ISI of 1500±500 ms. One half of the CSs was now paired with an electric shock (CS+), while the other half remained unpaired (CS−). For half of these 52 CS+, the electric shock was applied to the right index finger (CS+_right_), for the other half to the left index finger (CS+_left_). The electric stimulation appeared at 650 or 700 ms after picture onset. We used 100% contingent pairings, so that each CS+ was paired three times with the US (i.e., once during every learning run). All CSs were presented in a randomized order, with the only restriction that not more than three stimuli of the same condition followed each other. After the learning phase, stimulation electrodes were removed and participants completed the post-learning phase which, except for the differing stimulus randomization, was identical to the pre-learning phase. Before the pre- and the post-learning phases, participants were informed that they would see several different faces, but not receive any electric stimulation. To substantiate the subject’s confidence in this information, stimulation electrodes were attached after the pre- and detached before the post-learning phases. Before the learning phase, participants were informed that they would see several different faces and receive electric stimulation. They were, however, not informed about the presence of contingencies between the presentation of a face and the occurrence of an US.

Following the MEG measurement, participants repeated the valence and arousal SAM-ratings of all CSs and completed an affective priming task as well as a CS-US matching task (more detail provided below). The order of the SAM-rating and the affective priming task was balanced across participants to counteract systematic influences of extinction. The CS-US matching task was always performed last.

#### Affective priming task

The affective priming task consisted of 104 trials. On each trial, a fixation cross was shown for 200 ms and, then, a CS+ or CS− was presented for 200 ms. CS offset was immediately followed by a target word, to which participants made a binary forced choice valence decision (positive, negative). Half of the 52 target words were positive and half negative adjectives, matched in length, frequency, and arousal (word database from [27]). Every target word appeared twice, once paired with a CS+ and once paired with a CS− on first or second presentation (order counterbalanced across trials). Thus, prime face and target word were either *congruent* (threat-signaling CS+ preceding a negative adjective or safety-signaling CS− preceding a positive adjective) or *incongruent* (threat-signaling CS+ preceding a positive adjective or safety-signaling CS− preceding a negative adjective) with respect to the acquired hedonic valence. Participants were instructed to categorize the targets quickly and accurately as positive or negative, irrespective of the preceding face. For practice, they completed 15 test trials.

#### CS-US matching task

The CS-US matching task assessed whether participants were aware of the stimulus category (CS+ vs. CS−) and the shock location (left vs. right hand). Forty (10 CS+_right_, 10 CS+_left_, 20 CS−) of the 104 CS faces were pseudo-randomly selected for the task. Participants first viewed the CSs for 600 ms, preceded by a fixation cross shown for 2000 ms. Then, they indicated for each face whether it had been or had not been paired with an electric shock during conditioning (*rating of stimulus category*) and whether the electric shock had been applied to the right or the left hand (*rating of shock location*). For CS− categorized faces, participants were asked to guess a potential shock location under the prediction that they might have falsely categorized the face as unpaired. For practice, they completed three test trials.

Experimental stimulation during all phases was delivered using Presentation (Neurobehavioral Systems, Albany, CA, USA).

### Analysis of subjective and behavioral measures

#### Subjective ratings of valence and arousal

To assess whether conditioning changed evaluative judgments to aversively paired and unpaired faces, we compared the SAM-ratings acquired after to the ones acquired before MultiCS conditioning. Repeated-measures ANOVAs with the factors SESSION (pre-, post-conditioning) and CS-TYPE (CS+, CS−) were calculated across the hedonic valence and emotional arousal ratings.

#### Affective priming task

Trials with incorrect responses or reaction times (RTs) above or below two standard deviations of the individual mean were excluded from analysis, resulting in a rejection of 2.6% and 4.5% of the trials, respectively. In the present analysis, a different, more stringent criterion for exclusion of outliers was used as in the review article about MultiCS conditioning [16]. Due to these differences in the exclusion criteria, results of the affective priming task vary between reports. RTs were inverted to approximately fit a normal distribution and submitted to a repeated-measures ANOVA with the factors CONGRUENCY (congruent, incongruent), TARGET VALENCE (positive, negative), and REPETITION (first, second target presentation). In congruent trials (i.e., primes and targets share the same valence), responses should be facilitated and should occur with overall faster RTs than observed in incongruent trials (i.e., primes and targets are of opposing valence). Therefore, a significant effect of the factor CONGRUENCY (in the expected direction) would indicate a change in CS hedonic valence due to conditioning that is powerful enough to bias subsequent behavioral responses (e.g., [28]).

#### CS-US matching task

Trials in which participants did not make an explicit decision in favor of one of the two options (i.e., CS+/CS− or CS+_right_/CS+_left_) - although instructed otherwise - were excluded from analyses. The level of awareness concerning stimulus category (CS+ vs. CS−) and shock location (CS+_right_ vs. CS+_left_) was then assessed for each individual by calculating the sensitivity measure *d'* (e.g., [29]). In case of one participant, a false alarm rate of 100% was replaced by 97.5%, equivalent to (n−0.5)/n. For the assessment of stimulus category and shock location, statistical significance of *d'* values was evaluated by calculating a one-sample *t*-test of all individual *d'* values against zero.

Behavioral tests were analyzed using SPSS Statistics (IBM, Armonk, NY, USA).

### Preprocessing and analysis of MEG data

Visual evoked magnetic fields (VEMFs) were acquired using a 275 MEG whole-head sensor system (Omega 275; CTF Systems Inc.) with first-order axial SQUID gradiometers. The individual head position in the MEG scanner was tracked by three landmark coils placed on the two ear canals and the nasion. The individual head coordinate system was determined by a Polhemus 3Space Fasttrack which measured individual head shape information. VEMFs were recorded continuously in a frequency range between 0 and 300 Hz using a sampling rate of 1200 Hz. Offline, the recordings were sampled down to 600 Hz and filtered as to only include responses in a frequency range between 0.2 and 148 Hz. Filtering was conducted using zero-phase (forward and backward) Butterworth high-pass (second order) and low-pass (fourth order) filters. Epochs of 1000 ms duration (i.e., 200 ms before to 800 ms after CS onset) were extracted, aligned, and baseline-corrected using a 150 ms pre-stimulus interval as baseline which ranged from 150 ms before picture presentation to 0 ms (i.e., picture onset). Single trials were edited and artifacts were corrected following the method for statistical control of artifacts in high-density EEG/MEG data proposed by [30]. In this method, channels contaminated by artifacts are interpolated by weighted spherical splines fit to the neighboring sensors. When too many channels are contaminated within one trial, the trial is removed. The mean number of remaining trials did not differ between the experimental conditions [*Pre-learning vs. post-learning.* CS+_pre_: 146.4±6.3; CS−_pre_: 147±7; CS+_post_: 145.2±6.1; CS−_post_: 145±6.1; *F*(3, 188) = 1.1, *p* = .349. *Learning*. CS+_run1_∶48.2±3.1; CS−_run1_∶47.9±3.5; CS+_run2_∶48.6±3.1; CS−_run2_∶48.7±3.3; CS+_run3_∶48.5±3.1; CS−_run3_∶49.3±2.5; *F*(5, 282) = 1.22, *p* = .301]. Epochs were averaged in correspondence to the conditions. On the basis of the averaged responses, cortical sources of the event-related magnetic fields were calculated using the L2-Minimum-Norm-Estimates (L2-MNE) method [31]. The L2-MNE is an inverse modeling technique with which distributed neuronal network activity can be estimated. It does not require a-priori specifications of the location and/or number of active current dipoles [32]. We used a spherical shell with evenly distributed 2 (azimuthal and polar direction) × 350 dipoles as source model and chose a source shell radius of 87% of the individually fitted head radius which approximately corresponds to the gray matter depth. A Tikhonov regularization parameter *k* of 0.2 was applied across all participants and conditions.

Taking into account the individual sensor positions, topographies displaying the direction-independent current dipole activation were calculated for each participant, experimental condition, and time-point. The subsequent analysis consisted of two steps:

1. We investigated whether the SESSION × CS-TYPE interaction as reported in [16], which signaled a change in rapid CS processing after compared to before differential conditioning, would replicate under non-parametric test statistics and correction for multiple comparisons as proposed by [33]. To this end, a repeated-measures ANOVA with the factors SESSION (pre-, post-learning) and CS-TYPE (CS+, CS−) was performed for all modeled neural generators at the a priori defined time interval of interest 50–80 ms after CS onset. Analysis resulted in a distribution of statistical *F*-values for the main effects of SESSION and CS-TYPE and the interaction of SESSION × CS-TYPE for each dipole. Monte Carlo simulations of identical analyses based on 1000 random permutations of the complete data set of subjects and experimental conditions were conducted (SESSION × CS-TYPE interaction for each dipole within the 50 to 80 ms interval). We calculated first-level cluster statistics of a minimum of five neighboring dipoles achieving an alpha-level *p* = .05. Direction of effects within source regions surpassing a cluster-mass alpha-level of *p* = .05 were further explored using a continuative parametric SESSION × CS-TYPE ANOVA and post hoc paired samples *t*-tests.
2. We investigated whether the differential CS processing expressed by a SESSION × CS-TYPE interaction was a function of extinction (i.e., only present during the post-learning phase, but not during learning) or a function of associative learning (i.e., already visible during acquisition of CS+/US associations). Therefore, a repeated-measures ANOVA including the factors RUN (first, second, third learning run) and CS-TYPE (CS+, CS−) was calculated across the neural activation during the learning phase and again for all modeled neural generators at the a priori defined time interval of interest. Non-parametric cluster-level *F*-statistics, continuative parametric statistics, and post hoc paired samples *t*-tests were calculated corresponding to the above described procedure.

Preprocessing and analysis of MEG data was carried out using the Matlab-based EMEGS software [34]. Continuative analyses were conducted using SPSS Statistics (IBM, Armonk, NY, USA).

Greenhouse-Geisser corrected significance values are reported for all repeated-measures analyses, in which the assumption of sphericity was violated.

## Results

### Subjective and behavioral measures

#### Subjective ratings of valence and arousal

**Figure 2:**
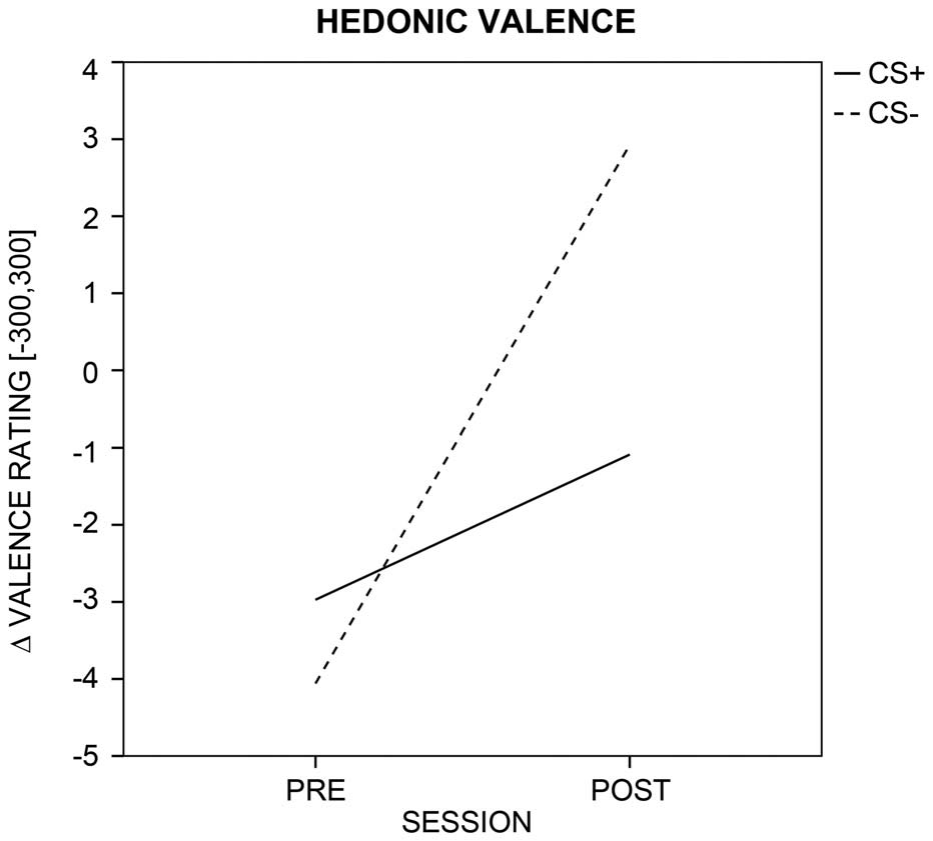
Evaluative results. Visualization of the change in hedonic valence for aversively paired (CS+; solid line) and unpaired (CS−; dotted line) faces across sessions (i.e., before and after MultiCS conditioning).

The repeated-measures ANOVA on the ratings of hedonic valence yielded a significant SESSION × CS-TYPE interaction (Figure 2; Dataset S1), *F*(1, 47) = 5.13, *p* = .028. Post-hoc *t*-tests showed that CS- stimuli were rated as more pleasant (by trend) after as compared to before conditioning (CS−_pre_: *M* = −4.06, *SD* = 32.73; CS−_post_: *M* = 2.92, *SD* = 29.71), *t*(47) = 1.98, *p* = .054, and that shock associated CS+ faces were rated as less pleasant (by trend) than CS− stimuli in the post-conditioning phase (CS+_post_: *M* = −1.09, *SD* = 30.00; CS−_post_: *M* = 2.92, *SD* = 29.71), *t*(47) = −1.83, *p* = .073. For comparison, an ANOVA on *z*-transformed valence ratings yielded also a significant SESSION × CS-TYPE interaction, *F*(1, 47) = 6.72, *p* = .013. Ratings of emotional arousal did not differ across SESSION or CS-TYPE, and there was no significant interaction (all *p*_s_ ≥.299).

#### Affective priming task

The repeated-measures ANOVA did yield neither the expected main effect of CONGRUENCY, *F*(1, 47) = 2.12, *p* = .152, nor an interaction of CONGRUENCY with any other factor (all *p*_s_ ≥.725; Dataset S2). There were only main effects of TARGET VALENCE, *F*(1, 47) = 76.83, *p*<.001, and REPETITION, *F*(1, 47) = 112.47, *p*<.001, as RTs were faster in trials in which target words were positive or presented for the second time.

#### CS-US matching task

Neither *d'* for the stimulus category nor *d'* for the shock location differed significantly from zero (*M* = 0.07, *SD* = 0.45, and *M* = 0.01, *SD* = 0.66, respectively), *t_s_*(47) = 1.10 and 0.08, *p_s_* = .275 and.937, indicating a lack of detectable contingency awareness for both stimulus category and shock location (Datasets S3, S4).

### Estimated neural activation

#### Post- versus pre-learning phase

**Figure 3:**
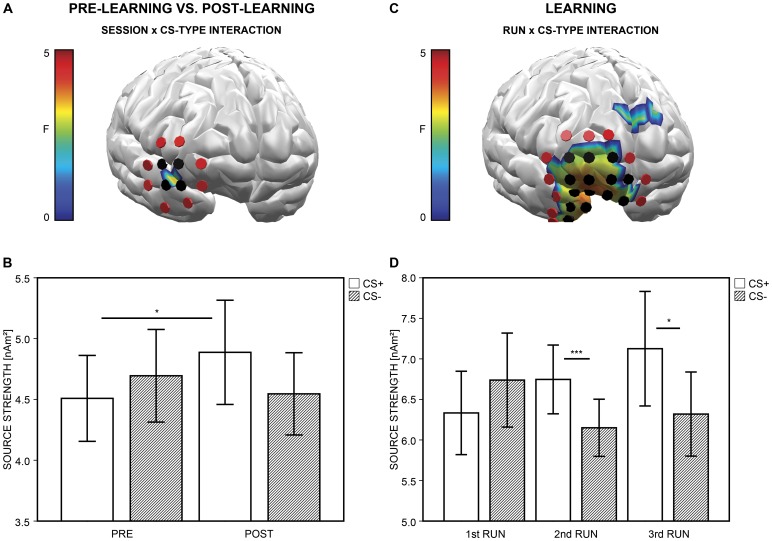
Neuronal activation. (A) Neuronal activation for paired (CS+) and unpaired (CS−) faces is compared between sessions (i.e., between the pre- and the post-learning phases). *F*-values (*p*<.05) for the SESSION × CS-TYPE interaction are projected onto a standard brain shown from right frontal view. Colored areas represent *F*-values significant with *p*<.05 based on the non-parametric statistical analysis. Black disks visualize the prefrontal test dipole locations used for the following post hoc parametric tests, while red disks visualize the actual extent on cluster level. (B) Bars depict the mean regional amplitude towards CS+ and CS− across sessions for the test dipole locations and the respective 95% confidence intervals. (C) Neuronal activation for CS+ and CS− is compared across the three runs of the learning phase. Coding of effects is identical to (A). (D) Bars depict the mean regional amplitude towards CS+ and CS− across the three runs of the learning phase for the test dipole locations and the respective 95% confidence intervals.

Analysis revealed a significant SESSION × CS-TYPE interaction in the right inferior frontal PFC during 50 to 80 ms (Figure 3A; Dataset S5), *F*(1, 47) = 4.66, *p* = .036. Indeed, CS+ activation increased from the pre- (*M* = 4.51, *SD* = 1.22) to the post-learning phase (*M* = 4.89, *SD* = 1.48), *t*(47) = 2.05, *p* = .046, and was enhanced (by trend) in comparison to CS− activation (*M* = 4.55, *SD* = 1.16) after learning (Figure 3B), *t*(47) = 1.93, *p* = .059. CS+ and CS− activation (*M* = 4.69, *SD* = 1.31) did not differ before learning, *t*(47) = −1.19, *p* = .242.

To explore whether effects were lateralized to the right inferior frontal PFC, we selected a left-hemispheric dipole group, homologous to the original right-hemispheric group, and calculated an ANOVA with the factors SESSION, CS-TYPE, and HEMISPHERE (right, left) across activation in both clusters during 50 to 80 ms (as suggested by [35]). Analysis yielded again a SESSION × CS-TYPE interaction, *F*(1, 47) = 6.54, *p* = .014, but the SESSION × CS-TYPE × HEMISPHERE interaction was not significant, *F*(1, 47) = 0.43, *p* = .516. Thus, although first-level cluster statistics revealed a two-way interaction only in the right inferior frontal PFC, continuative analyses suggest that effects were not lateralized to this region.

We observed a SESSION × CS-TYPE interaction during 50 to 80 ms also in the bilateral inferior occipital lobe and left temporo-parietal junction. As we were especially interested in learning-related changes in the PFC, these additional effects were not further investigated.

#### Learning phase

During the learning phase, we observed a RUN × CS-TYPE interaction again in the right inferior frontal PFC during 50 to 80 ms after CS onset (Figure 3C; Dataset S6), *F*(2, 94) = 5.69, *p* = .006. The interaction was mainly driven by a linear effect of the factor RUN on CS+ activation, *F*(1, 47) = 5.42, *p* = .024, and (by trend) by a quadratic effect of RUN on CS− activation (Figure 3D), *F*(1, 47) = 3.88, *p* = .055. Indeed, CS+ activation increased from the first (*M* = 6.33, *SD* = 1.77) to the third run (*M* = 7.13, *SD* = 2.43), *t*(47) = 2.33, *p* = .024, and CS− activation decreased from the first (*M* = 6.74, *SD* = 2.00) to the second run (*M* = 6.15, *SD* = 1.21), *t*(47) = −2.18, *p* = .034. Importantly, CS+ and CS− activation did not differ during the first learning run (i.e., before the first pairing), *t*(47) = −1.54, *p* = .130, but CS+/CS− differentiation was already enhanced after a single CS+/US pairing (i.e., during the second run), *t*(47) = 3.34, *p* = .002, and remained enhanced after two CS+/US pairings (i.e., during the third run), *t*(47) = 2.35, *p* = .023.

Consistent with the pre−/post-learning comparison, we investigated whether learning effects were lateralized to the right hemisphere, by selecting a left-hemispheric dipole group, homologous to the right prefrontal group, and computing an ANOVA with the factors RUN, CS-TYPE, and HEMISPHERE across activation in both clusters during 50 to 80 ms. A significant RUN × CS-TYPE, *F*(2, 94) = 4.14, *p* = .022, but no RUN × CS-TYPE × HEMISPHERE interaction, *F*(2, 94) = 1.06, *p* = .348, emerged. Thus, effects cannot be considered as being lateralized to the right PFC.

Analysis returned significant RUN × CS-TYPE interactions during the 50 to 80 ms time interval also in the right occipital and left middle temporal lobe. Since this study focused on early prefrontal activation during conditioning, these additional effects were not further explored.

## Discussion

Previous MultiCS conditioning studies reported enhanced PFC activation towards aversively paired vs. unpaired faces during 50 to 80 ms after as compared to before learning [16], [17]. Here, we showed that this differential PFC activation emerges under a non-parametric analysis procedure [33]. Furthermore, our results indicate that this differential PFC activation arises as a function of the acquisition and not the extinction of the CS+/US association. An analysis of the learning phase, in which multiple faces were paired with an aversive electric stimulation or remained unpaired, revealed increased PFC activation towards aversively paired stimuli during 50 to 80 ms already during the acquisition of CS+/US contingencies. This enhanced prefrontal response was visible after only a single pairing, which suggests that only one learning trial was sufficient to differentially modulate short-latency neuronal activation. Behavioral indices of learning indicated that MultiCS conditioning changes the subjective valence, but not the arousal of the conditioned stimuli. However, these slight changes in valence were presumably not powerful enough to influence response tendencies in an affective priming task. As participants could not differentiate between the stimulus categories (CS+ vs. CS−) or detect the shock location (right vs. left hand), we conclude that MultiCS conditioning occurred in the absence of contingency awareness.

### Subjective and behavioral correlates of MultiCS face-shock-conditioning

Unpaired faces were rated as more pleasant after compared to before learning, whereas pleasantness ratings of aversively paired faces did not change across sessions. This finding is in line with previous MultiCS conditioning studies in vision [17], [36], [37] and has been interpreted as a superposition of aversive conditioning and mere exposure [38]. Repeated exposure may lead to an increase in pleasantness ratings of all CSs independent of the US association, while the US association results in increased ratings for safety- and reduced ratings for shock-associated faces. The valence effects observed here are smaller compared to more traditional classical conditioning paradigms, in which only a single CS+ and a single CS− are used. These differences in effect size are most presumably the result of an explicit knowledge of the CS+/US association in traditional classical conditioning paradigms vs. a lack of this knowledge in MultiCS conditioning, as subjective ratings strongly rely on a cognitive evaluation of the CSs. Furthermore, it could be speculated that the differences in effect magnitude arise due to the fewer number of learning trials in MultiCS conditioning, which only induce a transient change in hedonic valence, and/or due to a regression to the mean as a result of the multitude of CSs.

The affective priming paradigm, with CS+ and CS− faces as primes to positive and negative words, did not show faster RTs in congruent compared to incongruent trials. This finding does not correspond to results of a previous MultiCS conditioning study in which an affective priming effect to emotional words was observed after the presentation of aversively paired (electric shock) and unpaired click-tones [39]. We speculate that the lack of an observable priming effect resulted from more volatile CS+/US associations in this compared to the previous study. As we used 104 CSs and three learning trials compared to 40 CSs and four reinforcement trials [39], the CS+/US associations in this study might have been too weak to bias a subsequent valence decision task.

Taken together, the findings suggest that MultiCS conditioning modifies behavior even under challenging conditions, which points to a great ability in humans to quickly acquire, preserve, and act on contingencies present in the environment. However, there are limits as to what extent such rather transient learning can influence action tendencies in an affective priming setting. As the third task failed to indicate awareness of CS+/US contingencies, the fast and highly resolving learning most likely occurred in the absence of contingency awareness.

### Affect-specific prefrontal modulation during and after learning

The PFC was differentially activated by aversively paired and unpaired stimuli already during the learning phase of MultiCS conditioning when a CS+/US association was being established. Importantly, this differential PFC activation during fear learning resembles in location and time of occurrence the differential PFC activation after fear learning as observed in the present and in previous MultiCS conditioning studies in vision [16], [17]. Such convergence supports the interpretation that enhanced PFC activation towards CS+ vs. CS− after learning (i.e., during unreinforced presentations of CS+ and CS−) reflects rather the access of the CS+/US association and thus the emotional value of the CSs than the extinction thereof. An involvement of the PFC in acquisition and retrieval of CS+/US associations appears somewhat inconsistent with the prevalent view that the PFC is mainly involved in the recall and the expression of extinction, but it is only assigned a minor role in the acquisition of fear memory (see reviews by [40]–[42]). This view is strongly influenced by classical conditioning studies with rodents. In humans, however, complex cognitive processes drive classical conditioning [43], which is why it should not come as a surprise that the PFC, as a hub for human cognition, is involved in human fear acquisition. Indeed, a recent meta-analysis [44] including fMRI studies on instructed and uninstructed (classical) conditioning revealed consistent PFC activation during fear acquisition in humans. The authors suggested several functions of the PFC during fear acquisition including the appraisal of threat, the learning of stimulus-reinforcer contingencies, and the generation of an adequate motor response. With regard to the present findings, we propose that the early PFC activation observed during fear acquisition represents the learning of CS+/US associations as well as subsequent threat appraisal and/or memory retrieval. Such rapid involvement of the PFC could facilitate the detection of potentially dangerous stimuli and bias following waves of sensory processing in the temporal cortex in favor of such threat-related stimuli (as for instance in [17]). This interpretation is supported by other considerations of PFC function in vision, under which the PFC has been connected to the representation of visual object categories [45] and to the top-down control of sensory and limbic areas [46], [47]. Of interest, it can be assumed that the high number of complex and similar stimuli used in the present task increased the difficulty of distinguishing between stimuli that signaled danger (CS+) and stimuli that signaled relative safety (CS−). Therefore, we speculate that such high task demands and the uncertainty of contingency strengthens the recruitment of higher-order areas (such as the PFC) to dissociate CS+ and CS− faces. Indeed, Bar and coworkers (2006) [48] observed rapid orbitofrontal cortex activation especially under those experimental conditions, under which object recognition is difficult. However, future studies using within-subject manipulations should further investigate whether the short-latency PFC activation during fear acquisition found here is enhanced under challenging experimental conditions.

### Neurophysiological model mediating short-latency prefrontal activation

The finding that aversive conditioning influences PFC activation during very early processing stages matches the results reported in visuo-olfactory MultiCS conditioning [17]. Drawing on the consistencies between the two studies, the present findings provide additional support for the hypothesis that emotion processing takes place in a highly resolving and rapid fashion involving higher-order cortical structures at an early stage of visual processing. With regard to the underlying neural mechanism, a neurophysiological model including multiple waves' of processing [49] and fast brain' structures [50] is best compatible with both the early latency of the present effects and their localization in the PFC. In such a model, visual information is initially processed along several parallel channels, resulting in multiple simultaneous sweeps of activation within the visual cortex and other brain areas [49]. An incoming visual stimulus reaches not only primary visual areas, but may also quickly engage other brain structures, such as the amygdala, insula, parietal, and frontal cortices [50], [51]. Via feedback-loops, these brain areas may provide top-down influence on the visual cortex, actively shaping on-going sensory processing [52], [53] and enhancing sensory representations in favor of appetitive and aversive stimuli, which are relevant to basic motivational centers in the brain [54]. A rapid transfer of visual information to various brain structures already during the initial wave of processing could take place via processing short-cuts [47], [49], [55]. Support for such short-cuts comes from studies recording extremely rapid response latencies (i.e., under 50 ms) to visual stimuli in sensory and prefrontal areas of the macaque and the human brain [50], [56], [57]. Additional evidence can be drawn from studies observing an influence of feedback connections on the response of primary visual areas within the first 100 ms of visual processing in nonhuman primate brains [58] or showing that frontal activation precedes activation in ventral visual areas [17], [48]. These findings support our interpretation of differential PFC activation at 50–80 ms to reflect first sweeps of visual processing that could exert top-down modulation on ongoing and/or subsequent sensory processing.

With respect to rapid PFC activation, we would like to note that differential processing observed in MultiCS conditioning matches findings of several studies investigating different types of learning other than classical conditioning. Using stimuli with targets at trained or untrained locations, it was recently shown that mere perceptual experience can modulate the C1 component as early as 44 ms after picture onset [59]. Similarly, stimuli with unconsciously predictive target locations were revealed to elicit enhanced neural responses between 50 and 100 ms [60], which subsequent intracranial recordings have suggested to be generated in the anterior and posterior temporal lobe and the orbitofrontal cortex [61]. Furthermore, learning to associate a certain facial feature and a personality trait has been observed to alter orbitofrontal, temporal, and inferotemporal responses towards novel faces already between 60 to 85 ms [62]. The spatiotemporal characteristics observed in this study appear thus as not specific for differential classical conditioning, because they show great similarity to findings of several different lines of research, including directed attention, perceptual learning, and implicit memory. Such a resemblance could suggest that the underlying neural systems might rely on similar mechanisms and even recruit overlapping neural circuits to enhance relevant sensory representations.

### Rapid plasticity in the PFC

Analysis of the learning phase indicated that one learning trial was sufficient to evoke differential PFC activation. Single-trial learning in classical conditioning is a quite controversial phenomenon. The most compelling evidence arguing for the existence of single-trial learning comes from studies that demonstrate the acquisition of taste aversion in rodents and humans after only one bad experience with certain foods [63] (see reviews [64], [65]). Single-trial classical conditioning using electric stimulation and appetitive foods as USs has also been observed in more simple organisms, as in the aplysia californica and the snail lymnaea stagnalis [66]–[68]. In humans, experimental evidence for the acquisition of conditioned responses in a single learning trial is scarce. Öhman and colleagues (1975) [69] showed that enhanced skin conductance responses towards aversively paired conditioned stimuli are similarly resistant to extinction after five and after only a single learning trial, when phobic stimuli are used as CSs. In a classical conditioning setting, Weisz and coworkers (2007) [70] demonstrated differential responses in the auditory cortex within the first five learning trials, which distinguished between information that was important and not important to the task. Yet, other reports suggest that more CS+/US pairings are necessary to evoke a neuronal CS+/CS− differentiation [71], [72]. This divergence of results could suggest that single-trial learning can only be observed under certain conditions, such as when certain types of USs, certain combinations of CSs and USs, or certain response measures are used. However, certain characteristics of the MultiCS conditioning paradigm might have enabled us to observe differential neuronal CS+/CS− responses after a single trial of acquisition. Especially the high number of CS+ and CS− faces increased the signal-to-noise ratio, due to which small changes in activation could have been made visible.

Apart from classical conditioning, single-trial acquisition is also known for learning of novel stimuli. For instance, Rutishauser and coworkers [73] showed that different types of neurons in the human hippocampus and amygdala reacted with increased firing rate towards novel and familiar complex pictorial stimuli after a single preceding stimulus presentation. Similarly, Salzmann and coworkers [74] reported a change in firing rate of neurons in the non-human primate amygdala within the first trial of reversed stimulus-reinforcement contingencies.

Importantly, increased firing rates in amgydala neurons or enhanced activity within the inferior lateral PFC – which is closely connected to the amygdala (e.g., [74]) - seem to reflect an automatic identification of stimulus saliency which might be a necessary but definitely not sufficient condition for contingency awareness or adaptation of behavior. It is quite likely that information from multiple brain areas and multiple processing stages is necessary for a stimulus contingency to flood into consciousness or to modulate behavior. Dissociations between rapid correlates of saliency identification and behavior might – at least with regard to these early phases of learning – be thus rather the rule than the exception.

### Limitations

At last, we would like to point out that the present study has limited informative value with regard to revealing the exact spatial location and regional extent of the differential PFC activation. This limitation arises as consequence upon the choice of the inverse method used here. Assuming that our target region in the inferior frontal cortex is just one of multiple nodes in a distributed and simultaneously active network, we chose a distributed source reconstruction with Minimum Norm Estimate. This method approximates the spatial location of cortical generators and allocates these generators to larger cortical structures fairly well, but identification of distinct Brodman areas would - with respect to the approximated norm - in this special case pretend false accuracy. Furthermore, the Minimum Norm method puts specific emphasis on distributed cortical sources which is why inferences about the regional extent of the underlying neuronal activity cannot be made.

### Conclusions

MultiCS conditioning is a challenging and highly resolving learning paradigm. It induces changes in subjective ratings towards the conditioned stimuli that are typically observed during classical conditioning. It is accompanied by rapid neuronal plasticity in the PFC after only a few learning trials, visible in enhanced neuronal activation towards paired stimuli at short-latency processing stages during and after learning. Due to the multitude of stimuli in one condition (paired vs. unpaired), it offers a high signal-to-noise ratio and is thus well suited for EEG/MEG research. Moreover, it seems to be an ideal method for visualizing fast implicit learning processes in the brain. Since it encompasses a relatively weak and ambiguous learning situation in which CS+/US associations are not clear, it could aid in uncovering individual differences between patient and control populations as well as subclinical risk- and non-risk groups [75]. In this matter, MultiCS conditioning could be especially useful in the research of anxiety disorders, such as post-traumatic stress disorder, which can arise due to the experience of a single traumatic event [76].

## Supporting Information

Dataset S1 **Dataset underlying analysis of subjective ratings of valence and arousal.** The spreadsheet contains original (“Response_mean”) and *z*-transformed (“zscores_mean”) ratings of valence (“val”) and arousal (“aro”) for all 48 participants aggregated by session (“pre”/“post”) and CS-type (“plus”/“minus”).
(XLSX)

Dataset S2 **Dataset underlying analysis of the affective priming task.** The spreadsheet contains original (“RT_mean”) and inverted (“ONEdivbyRT_mean”) reaction times for all 48 participants aggregated by congruency (“congr”/“incongr”), target valence (“pos”/“neg”), and repetition (“first”/“sec”).
(XLSX)

Dataset S3 **Dataset underlying analysis of the CS-US matching task (stimulus category).** The spreadsheet contains the total number of hits (“hit”), misses (“miss”), false alarms (“falsealarm”), correct rejections (“correctrejection”), signal trials (“total_CSplustrials”), and noise trials (“total_CSminustrials”) for all 48 participants. It additionally displays the hit (“hitrate”) and false alarm rates (“falsealarmrate”), the Z values for the hit (“z_hitrate”) and false alarm rates (“z_falsealarmrate”), and the final *d'* value (“dprime”).
(XLSX)

Dataset S4 **Dataset underlying analysis of the CS-US matching task (shock location).** Labeling of variables as in S3, except that *d'* is labeled as “dprime_shockLocation”.
(XLSX)

Dataset S5 **Dataset underlying analysis of neuronal activation during the post- versus pre-learning phase.** The spreadsheet contains the neuronal activation of all 48 subjects in the right (“Right”) and homologous left-hemispheric (“Left”) dipole groups for the different sessions (“pre”/“post”) and CS-types (“plus”/“minus”) averaged across the 50 to 80 ms time interval.
(XLSX)

Dataset S6 **Dataset underlying analysis of neuronal activation during the learning phase.** The spreadsheet contains the neuronal activation of all 48 subjects in the right (“Right”) and homologous left-hemispheric (“Left”) dipole groups for the different runs (“R1”/“R2”/“R3”) and CS-types (“plus”/“minus”).
(XLSX)
